# Speciation History Shapes Patterns of Assemblage Species Richness in Birds

**DOI:** 10.1111/ele.70432

**Published:** 2026-06-22

**Authors:** Bouwe R. Reijenga, Rampal S. Etienne, David J. Murrell, Alex L. Pigot

**Affiliations:** ^1^ Department of Earth Sciences University of Oxford Oxford UK; ^2^ Centre for Biodiversity and Environment Research, Department of Genetics, Environment and Evolution University College London London UK; ^3^ Groningen Institute for Evolutionary Life Sciences University of Groningen Groningen the Netherlands

**Keywords:** coexistence, colonisation, community assembly, ecological limits, species richness, sympatry, time‐for‐speciation

## Abstract

Speciation is the ultimate source of biodiversity, yet because most species arise in spatial isolation (allopatry), it remains unclear how speciation history shapes patterns of sympatric species richness. Here, we examine how the timing of past speciation events influences the maximum sympatric species richness attained across 40 families of passerine birds. Using a phylogenetic model, we infer that the average waiting time for species to assemble in sympatry is remarkably long (~8 million years), occurring over macroevolutionary timescales comparable to the pace of speciation itself. Consequently, we find that the proportion of species in sympatry varies substantially across families, peaking in ancient or small clades comprised of older species, while remaining low in large, rapidly diversifying clades. Our analysis shows that macroevolutionary delays in colonisation are sufficient for speciation history to leave an indelible legacy on present‐day assemblages, challenging the view that richness is strictly limited by contemporary environmental capacity.

## Graphical abstract

Speciation is the ultimate source of biodiversity. However, because most species arise in spatial isolation, how speciation shapes patterns of co‐occurring species richness remains unclear. Here we examine how the legacy of speciation affects contemporary biodiversity patterns via a phylogenetic model and show that this build‐up is remarkably slow and challenges views on strict limits to richness by contemporary environmental capacity.

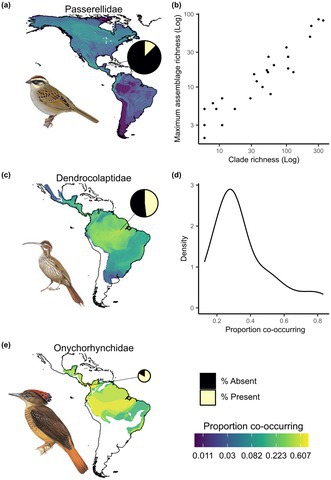

## 1 Introduction

The extent to which the number of species found at a particular place on the Earth's surface reflects current environmental capacity or the legacy of history remains a central debate in ecology (Francis and Currie 2003; Harvey et al. 2020; Jetz et al. 2004; Jiménez‐Alfaro et al. 2018; Ricklefs 1987; Sonne and Rahbek 2024). While the correlation between environment and richness is well‐documented, focus has increasingly turned to how history influences species assemblages (Carrasco et al. 2022; Stephens et al. 2025). In some cases, the recency of major disturbance events allows the effect of history to be observed directly, such as the recolonisation of plants and animals following the 1883 eruption of Krakatau (Bush and Whittaker 1991) or the retreat of northern hemisphere ice sheets at the end of the Pleistocene (Blois et al. 2010). Over deeper timescales, where community assembly must be inferred, phylogenetic approaches have been used to model the dynamics of species diversification and dispersal within and among biomes, regions and islands (Jetz and Fine 2012; Kozak and Wiens 2012; Miller et al. 2018; Quintero et al. 2023; Ricklefs and Bermingham 2001; Stephens and Wiens 2003; Swiston and Landis 2025; Valente et al. 2020; Wiens 2011). While this previous research has advanced our understanding of how species pools expand within large, geographically isolated regions, these studies do not model how rapidly species accumulate locally, within the finer spatial grains (e.g., ~25–100 km grid cells) at which broad‐scale gradients in species richness are typically mapped (Orme et al. 2005; Rahbek and Graves 2001). Understanding how history shapes the accumulation of species richness at these finer spatial scales, requires consideration of both species origination and the rate at which these lineages assemble at a given location.

The origin of most species involves some form of spatial separation (allopatry or parapatry) that reduces gene flow between populations (Coyne and Orr 2004; Phillimore et al. 2008). As a result, cladogenesis becomes increasingly unlikely in geographic areas that are small relative to the spatial scale of dispersal (Coyne and Price 2000; Kisel and Barraclough 2010). In birds, for example, cladogenetic speciation is extremely rare or debatable in continuous areas smaller than Madagascar (1600 × 350 km) (Coyne and Price 2000; Phillimore et al. 2008). The richness of local assemblages—here defined as the species co‐occurring at a spatial grain below the limit of cladogenesis—is therefore generated by colonisation from a regional pool and eroded by local extinction (MacArthur and Wilson 1967; Mittelbach and Schemske 2015; Pigot and Etienne 2015). Although speciation does not directly add species to assemblages, it shapes their richness indirectly by structuring the available pool. First, the cumulative number of speciation events will influence the size of the species pool from which local assemblages can be formed. Second and more rarely addressed, the temporal distribution of these events will determine the age of the species in the pool and thus the time available to colonise local assemblages.

The significance of this temporal window is highlighted by empirical evidence suggesting that the transition from allopatry to sympatry required for the local build‐up of richness is a remarkably long process. Indeed, most sister species retain non‐overlapping distributions for millions of years following the initiation of speciation (Alencar and Quental 2023; Anderson and Weir 2022; Pigot and Tobias 2015; T. D. Price 2010; Weir and Price 2011). This delay often begins with the geographic features that promoted speciation, such as mountain ranges or rivers, which can persist as barriers to dispersal long after speciation is completed (Naka and Brumfield 2018). Even if these barriers are surmounted, sympatry can be blocked by incomplete reproductive isolation, leading to persistent hybrid zones or lineage fusion (Cooney et al. 2017; Price and Bouvier 2002). Furthermore, establishing widespread co‐occurrence may depend on ecological compatibility, requiring divergence in resource‐related traits (e.g., beak size) to overcome competitive exclusion (Anderson and Weir 2021; Pigot and Tobias 2013; Reijenga et al. 2023). These barriers—ranging from physical isolation to reproductive and ecological interference—are not mutually exclusive and can act in concert to delay the colonisation of assemblages and preserve the imprint of speciation history.

Regardless of the specific mechanisms limiting successful colonisation, the time elapsed since speciation represents a potentially fundamental constraint on local species richness. Specifically, when speciation events occur deeper in time, lineages have a longer temporal window to overcome the intrinsic and extrinsic barriers to sympatry, thereby increasing assemblage richness (Stephens and Wiens 2003). Given the existence of strong geographic and clade‐level gradients in rates of speciation (Harvey et al. 2020; Jetz et al. 2012; Rabosky et al. 2018; Schluter and Pennell 2017), variation in the timing of past speciation events could be an important driver of gradients in contemporary richness. Yet, the extent to which ‘time for colonisation’ limits the richness of local assemblages and can explain differences in sympatric diversity remains unquantified.

A scenario in which ‘time for colonisation’ constrains assemblage species richness bears similarity to the ‘time for speciation’ effect (Hutter et al. 2013; Stephens and Wiens 2003), but there are critical distinctions. Whereas ‘time for speciation’ typically explains the size of the regional pool by the duration of diversification (Wiens et al. 2011), ‘time for colonisation’ focuses on how the timing of speciation events influences how many of these species are able to assemble locally. Furthermore, while ‘time for speciation’ is often framed as a historical alternative to ‘ecological limits’ (Wiens 2011), in which richness is regulated around an equilibrium set by competition for finite resources (Etienne and Haegeman 2012; Phillimore and Price 2008; Rabosky 2009), the ‘time for colonisation’ effect is mechanistically linked to both. Ecological processes, such as competition, may provide the very barriers that slow the attainment of sympatry (Anderson and Weir 2022; Price et al. 2014). However, if these barriers take millions of years to overcome (Pigot and Tobias 2013), then local richness will be primarily limited by the recency of speciation rather than a fixed environmental carrying capacity. In this view, history and ecology are not mutually exclusive as ecological interactions serve to preserve the imprint of speciation history by slowing the rate at which assemblages reach equilibrium.

**FIGURE 1:**
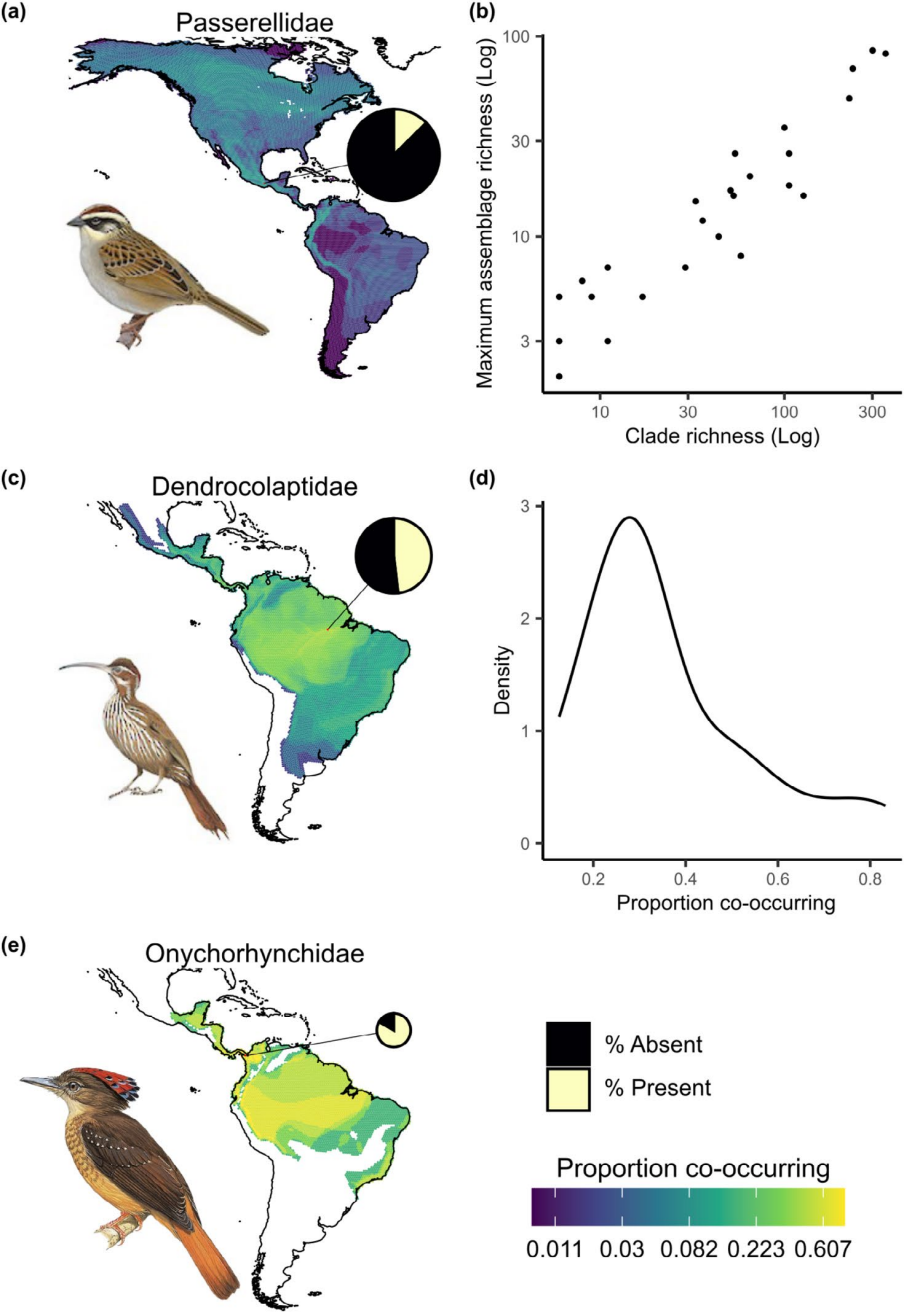
Species richness patterns of exemplary clades. (a, c, e) Assemblage species richness (as a proportion of clade richness) of three exemplar avian families showing low, intermediate and high levels of co‐occurrence. The size of the pie charts shows clade richness and the black lines point toward the grid cell of highest richness. (b) The relationship between clade richness and maximum local species richness (*n* = 40 families). An ordinary least squares regression estimates an intercept of −0.257 and slope of 0.761. (d) Distribution of maximum (proportional) local species richness across clades.

Here we develop an approach to test the extent to which speciation history—and the resulting time available for colonisation—predicts the build‐up of local species richness across 40 family‐level clades of primarily North and South American passerine birds. Because the richness of local assemblages is necessarily constrained by clade size (Figure S1) and this effect of pool size has been widely studied (Kennedy et al. 2017, 2018; Kozak and Wiens 2012; Weir 2006), we focus on explaining the maximum proportion of species in each clade found in sympatry. These passerine radiations are ideal in this respect because they differ widely in both their phylogenetic branching patterns (i.e., speciation history) which should result in predictable differences in sympatry and the maximum proportion of species that co‐occur—ranging from 12.8% in Passerellidae to 83.3% in Onychorhynchidae (Figure 1).

Our analysis consists of the following key steps. First, for each family we use geographic range maps to characterise assemblages of sympatric species within hexagonal grid cells spanning ~55 km in width. This grid cell size corresponds to an area substantially smaller than the minimum scale of speciation in birds allowing us to assume that new species are added to assemblages exclusively through colonisation. We focus on the most speciose assemblage within each family, the location of which can and often does, vary across clades. Second, given the set of species present in each of these focal assemblages and the speciation history captured by the phylogenetic branching patterns of each clade, we use a maximum likelihood‐based framework to estimate the rates of colonisation γ and local extinction μ that best explain the present‐day composition of these assemblages. Our framework uses the Dynamic Assembly Model of Colonisation, Local Extinction and Speciation, ‘DAMOCLES’ (Pigot and Etienne 2015) that has previously been applied to infer the dynamics of community assembly within individual clades, but here we extend this to model community assembly across multiple clades simultaneously. While DAMOCLES has mainly been used as a null model for detecting non‐stochastic community assembly processes (e.g., environmental filtering or competition) (García‐Navas et al. 2023; Marx et al. 2017; Pigot and Etienne 2015; Pinto‐Ledezma et al. 2019), here we instead apply this approach to infer γ and μ and are agnostic to the factors (e.g., biotic interactions or dispersal etc.) that regulate these dynamics. We note that without a complete fossil record for each assemblage, the timepoint at which each species colonised these locations is unknown. However, given the large spatial scale of speciation relative to our assemblages, we can be confident that following each speciation event, at least one of the daughter species must initially have been locally absent. DAMOCLES uses this insight to constrain when colonisation could have occurred and to estimate the rate that best explains current assemblage structure. Third, we use our estimates for γ and μ to stochastically simulate colonisation and local extinction events over the history of each clade and generate expectations for the current local assemblage richness attained by each family. In our main model we constrain γ (and μ) to be identical across all clades, so that any differences in expected assemblage richness are due solely to differences in the speciation history of each clade. In this way, we test how well speciation history can predict observed variation in assemblage species richness across clades, as well as how assemblage species richness varies with a suite of phylogenetic metrics describing the historical dynamics of speciation. Throughout, we compare the fit and predictive ability of this model, assuming a single universal rate of γ (and μ), to a model where these parameters can vary independently across families, to test how relaxing our assumption of equivalent rates alters our conclusions. Finally, we compare this model to a scenario where γ is fixed at a very large value and only μ is estimated. In this artificial scenario, the colonisation of species assemblages occurs almost instantaneously relative to the timescale between speciation events and so speciation history has no effect on current assemblage composition or richness.

## 2 Materials and Methods

### 2.1 Phylogenetic and Geographic Range Data

We compiled a dataset consisting of oscine and suboscine passerines predominantly endemic to the Americas. These clades were chosen because their high species richness (*n* = 2081 species) provides sufficient statistical power and because phylogenetic and geographic data are nearly complete. Evolutionary relationships and divergence times were obtained from two time‐calibrated phylogenies containing > 95% (oscines) and > 98% (suboscines) of described extant species (Barker et al. 2015; Harvey et al. 2020). To examine how clade‐specific speciation histories impact present‐day assemblage richness, the phylogenies were subdivided into family‐level clades with at least five species, resulting in 7 oscine and 18 suboscine clades. This balanced sufficient variation in patterns of assemblage richness with large enough clades to reliably calculate phylogenetic metrics.

To identify the species co‐occurring in each family we used expert delineated range maps (BirdLife International and Handbook of the Birds of the World 2020). Compared to field inventories which are often incomplete, range maps provide a comprehensive characterisation of species richness patterns albeit at a coarser spatial resolution. Because the phylogenies and range maps were based on different taxonomies, we aligned them by using the phylogenetic taxonomy and merged or split species geographic ranges accordingly. Range maps were then projected onto an equal area hexagonal grid (~2600 km^2^ per cell), where each hexagon has a side length of ~31.6 km and the distance between cell centroids is ~55 km (Barnes and Sahr 2017). This grain size is comparable to those used in macroecological analyses (Jetz and Fine 2012; Pigot et al. 2016; Rahbek 2005) and is much smaller than the minimum area required for in situ speciation to occur in birds (Coyne and Price 2000; Kisel and Barraclough 2010), thus ensuring that co‐occurring species arrived through colonisation. For each family‐level clade, we identified the grid cell of maximum species richness and recorded its composition. For the small number of species lacking expert range maps (*n =* 5, equally distributed among clades of > 53 species each), we used auxiliary information (e.g., occurrence records) to confirm their presence/absence in the grid cell of maximum richness for each family. While acknowledging that expert range maps are subject to errors of commission at small grain sizes (Hurlbert and Jetz 2007), we repeated our analyses using a finer grid (~96 km^2^ area with cell centroids ~10 km apart and sides of ~6 km) that reduces the chance that allopatric species separated by narrow geographic barriers are scored as co‐occurring.

### 2.2 Estimating the Dynamics of Community Assembly

**FIGURE 2:**
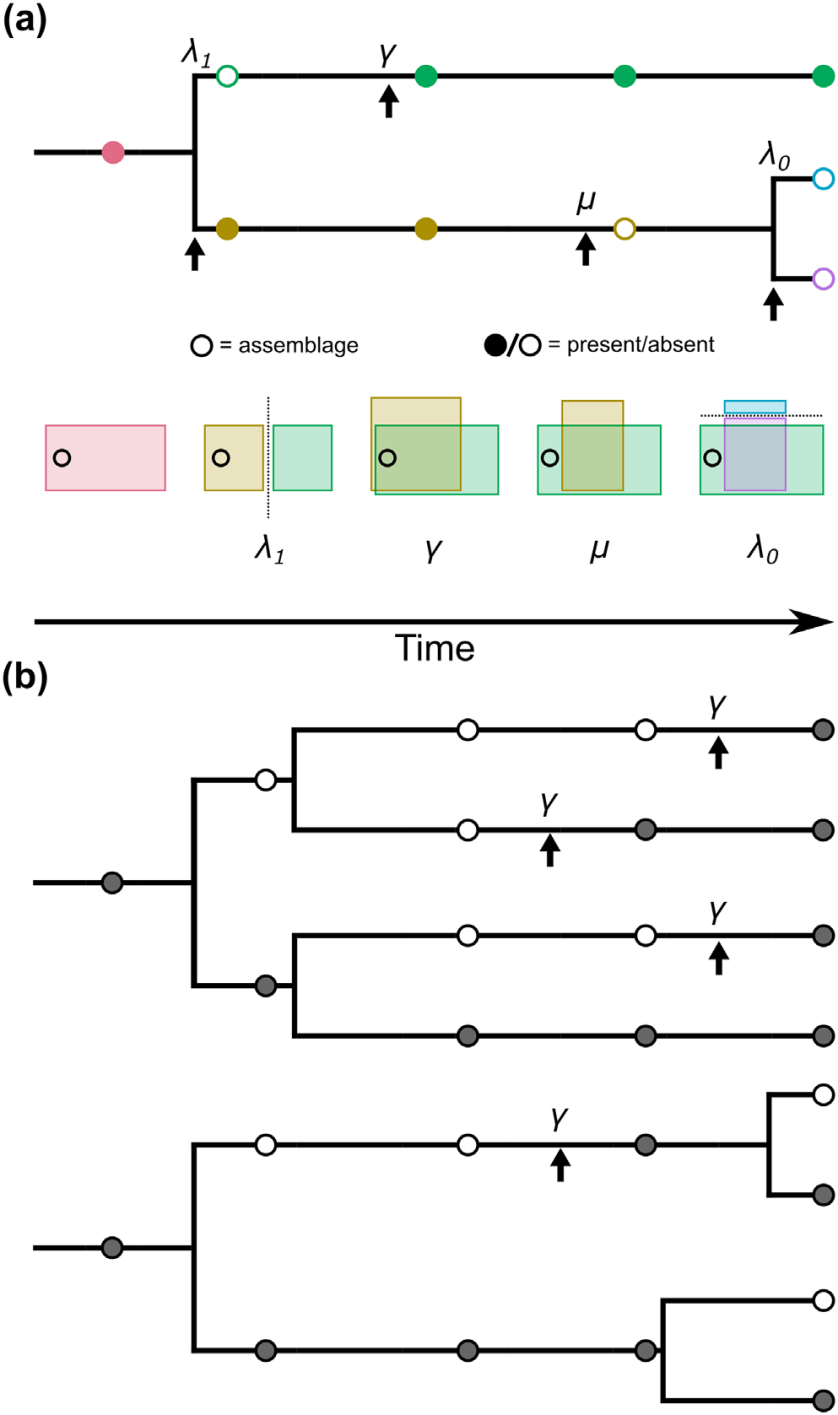
Visualisation of how speciation history influence assemblage richness. (a) Conceptualisation of the link between allopatric speciation, range expansion and assemblage richness. Upward‐pointing arrows show timing of events. When speciation occurs the ancestral geographic range (red rectangle) is divided into two (yellow and green) and at most one species will be present (λ_1_) in the local assemblage (circle). If the ancestor is not present, neither of the descendants (blue and purple) will be present (λ_0_) in the local assemblage. The build‐up of local assemblage thus depends on the colonisation of species (γ), as well as local extinction (μ), as species undergo expansion (green) or contractions (yellow) of their geographic range. (b) Two hypothetical clades, each with four species, with the top clade having older species on average than the bottom clade. Filled circles highlight the presence of a lineage in the local assemblage and arrows indicate colonisation events. For both clade the ancestor was present in the assemblage, but the dynamics of allopatric speciation and colonisation result in a higher assemblage richness at present in the clade with older species.

To test if speciation legacies can explain differences in assemblage richness across clades we applied and modified the DAMOCLES framework (Pigot and Etienne 2015). DAMOCLES models the assembly of a single community from a dynamically evolving regional pool via the processes of colonisation (γ) and local extinction (μ) (Figure 2a), assuming constant rates through time and among lineages. Speciation is not modelled. Instead, the composition of the regional pool at time *t* reflects the lineages in an empirical reconstructed phylogeny that are extant at time *t*. DAMOCLES assumes that cladogenesis will result in one daughter lineage being present and one absent if the parent lineage is present in the assemblage and that both will be absent if the parent is absent (Figure 2b). Given this model, the phylogeny and a vector of the presence (1) and absence (0) of each species in the grid cell of maximum richness, we estimated *γ* and *μ* using maximum‐likelihood.

Obtaining the likelihood of an assemblage (i.e., the vector of presence/absence) resembles the Felsenstein pruning algorithm (Felsenstein 1981), detailed in Pigot and Etienne (2015). Operating from extant tips to the root, we track each lineage back in time, conditioning on observed presence (1) and absence (0) at the tips. We define $p_{\text{if}}\left(t\right)$ as the probability that the descendants of a lineage are in state *f* at the present (time 0), given that the lineage was in state *i* at time *t* in the past. The change in these probabilities along a branch is described by a system of Ordinary Differential Equations (ODEs), which are integrated backward in time until a node is reached. At each node, probabilities of the two descendant lineages are combined and the two lineages are pruned. This process is repeated until there are no more branches left to prune. The likelihood at the root is calculated by combining *p*_*0f*_ and *p*_*1f*_, with the combination determined by the prior distribution on the root state. Assuming complete ignorance, we compute the likelihood as the sum of these probabilities. The values of γ and μ that best explain a community are obtained by maximising this likelihood. To ensure robust inference, we fitted models by using multiple solvers to confirm numerical stability, using multiple starting parameter values and multiple optimisation algorithms to ensure convergence to the global likelihood maximum.

### 2.3 Testing the Effect of Speciation History on Assemblage Richness

We tested the effect of speciation history on maximum assemblage richness across clades via three model scenarios. First, a ‘historical global rate’ model estimated a single γ and μ across all clades, such that differences in (proportional) assemblage richness across clades arise entirely from differences in speciation history and time for colonisation. Second, a ‘historical variable rate’ model estimated γ and μ separately for each family, thus relaxing the assumption that these dynamics are identical across clades. For both scenarios, the likelihood was conditioned on assemblages containing at least one species. Finally, we evaluated a ‘non‐historical’ null model only estimating μ and fixing γ at a very high rate (γ = 1000), representing effectively no lag time to colonisation after speciation. Failure to reject this null model would indicate no detectable effect of speciation history on the richness of species assemblages. We compared the fit of the ‘historical global rate’, ‘historical variable rate’ and ‘non‐historical’ model using AIC. Parametric bootstrapping was used to estimate the 95% CI for the ‘historical global rate’ and ‘historical variable rate’ model parameter estimates (Table S1).

We assessed model adequacy of the ‘historical global rate’, ‘historical variable rate’ and ‘non‐historical’ scenarios by comparing their ability to reproduce the observed assemblage richness of each family and also to predict how assemblage richness varies according to different phylogenetic metrics describing the history of speciation within each clade. For each model, we simulated lineage colonisation and local extinction of assemblages for each family using estimated γ and μ values. Simulations were implemented via a Gillespie algorithm (Gillespie 1977) and modelled from the root of the family phylogeny at *t* = 0 until the present. Lineages transitioned from being absent (0) to being present (1) via colonisation (0 → 1) and local extinction (1 → 0) events, assigning the root state with equal probability. Waiting times between events (δ) were drawn from an exponential distribution based on the summed per‐lineage rates. At time *t* + δ, the event type and undergoing lineage was drawn based on the individual rates. This framework was already incorporated in DAMOCLES, but we re‐wrote this in *Rcpp* (Eddelbuettel and Balamuta 2018). Simulations were repeated 2500 times per clade to obtain the mean and 95% confidence interval (95% CI) in simulated assemblage richness under the ‘historical global rate’, ‘historical variable rate’ and ‘non‐historical’ scenarios.

We used the 95% CI in simulated assemblage richness to identify families with significantly higher or lower assemblage richness than expected under each model. We then tested how both the observed and mean simulated assemblage richness varied across clades according to the following phylogenetic metrics. For each family we quantified: (i) the crown age (Myr), (ii) tree imbalance quantified using Colless' index standardised for tree size and richness, where higher values indicate greater imbalance (Bortolussi et al. 2006; Mooers and Heard 1997), (iii) diversification (speciation—extinction) rate change (ρ), which measures slowdowns (ρ < 0) or speed‐ups (ρ > 0) in diversification through time (Etienne and Rosindell 2012; Janzen and Etienne 2024; Pigot et al. 2010) and (iv) mean terminal branch length (mbl) as a measure of the average age of extant species. Each metric was tested as a predictor of proportional assemblage richness across families using quasibinomial GLMS with a logit link (Douma and Weedon 2019). We fit separate models for the observed assemblage richness and the mean simulated richness for each of our three scenarios (i.e., ‘historical global rate’, ‘historical variable rate’ and ‘non‐historical’). Analysis was restricted to clades with more than five species, because calculating phylogenetic properties for very small trees becomes less informative.

## 3 Results

### 3.1 The Dynamics of Community Assembly

According to the ‘historical global rate’ model, the maximum likelihood per lineage rate of colonisation and local extinction is γ = 0.120 Myr^−1^ and μ = 0.178 Myr^−1^ respectively. This equates to a mean lag time from speciation to the colonisation of the focal assemblage of 8.3 Myr. A similar dynamic is inferred using the ‘historical variable rate’ model. In the ‘historical variable rate’ model, estimates of γ (0.04–100 Myr^−1^) and μ (0–187 Myr^−1^) vary substantially across different families (Table S1). However, families with very high estimated rates of γ and μ are small and thus have low information content such that rate estimates are highly uncertain (Table S1). As a result, the mean lag‐time to colonisation weighted by clade richness under the ‘historical variable rate’ model (7.9 Myr) is similar to that of the ‘historical global rate’ model (8.3 Myr). Comparison based on AIC indicates that the ‘historical global rate’ model (AIC_*γμ*_ = 2333.398), assuming a single γ and μ across all clades (*n* = 2 parameters), is better supported than the more parameter‐rich ‘historical variable rate’ model (AIC_*V*_ = 2350.29, *n* = 50 parameters, corresponding to separate γ and μ for each clade). Importantly, both the ‘historical global rate’ (AIC_*γμ*_ = 2333.398) and ‘historical variable rate’ model (AIC_*V*_ = 2350.29) provide a substantially better fit to the data than a ‘non‐historical’ model in which rapid colonisation and local extinction dynamics erase the effects of speciation history (AIC_*NH*_ = 2379.482). While exact rate estimates vary, these conclusions were consistent when using a finer grain size to define species assemblages (96 km^2^, Figures S2 and S3) and when selecting focal assemblages matching the mean level of richness observed within each clade rather than the maximum species richness (Figure S5).

### 3.2 The Effect of Speciation History on Assemblage Richness and Phylogenetic Metrics

**FIGURE 3:**
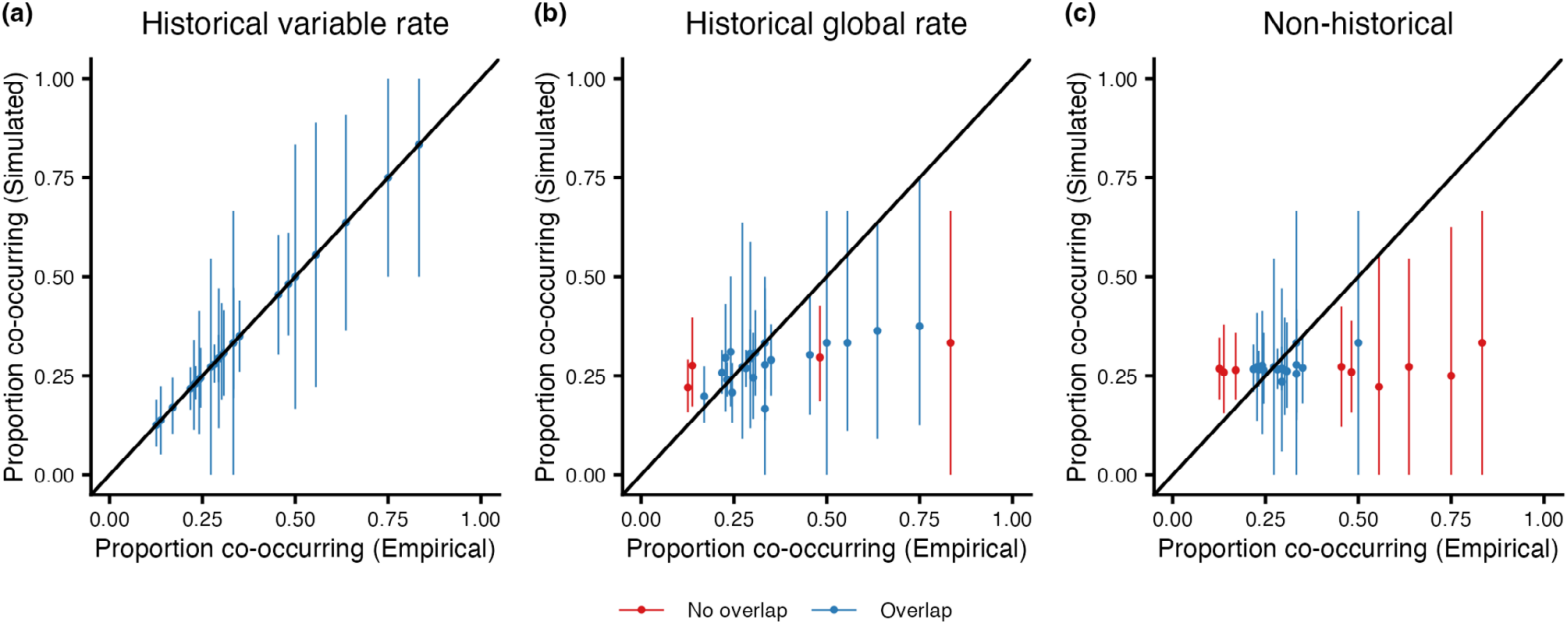
Observed variation in assemblage richness of passerine clades compared to model predictions. Assemblage richness is plotted as a % of the total number of species in each clade. Model predictions are shown for (a) the ‘historical variable rate’, (b) ‘historical global rate’, and (c) the ‘non‐historical’ model. The 1:1 line shows where the empirical the predicted richness matches. Bars represent the 95% confidence intervals of predicted assemblage richness from 2500 replicate simulations of each model. Colours indicate if empirical assemblages fall within (blue) or outside (red) of model expectations.

**FIGURE 4:**
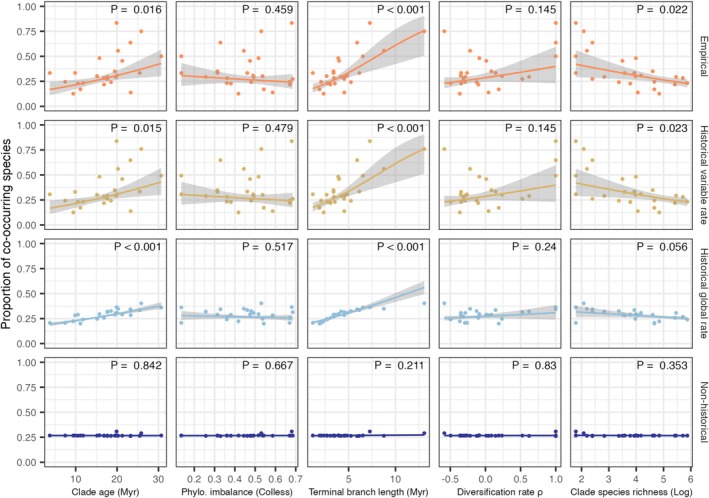
Relationship between phylogenetic metrics and assemblage species richness for real bird clades and model simulations. Colours and rows denote the empirical relationships for all clades (orange) and relationships simulated under the ‘historical variable rate’ (yellow), ‘historical global rate’ (blue) colonisation rate and ‘non‐historical’ (dark blue) model. For the simulation scenarios the mean proportion of co‐occurring species across 2500 replicated simulations are plotted. Columns represent the five phylogenetic metrics capturing different aspects of clade evolutionary history: The crown age of the clade, phylogenetic imbalance (Colless' index), mean branch length of the extant species, ρ a measure of temporal change in diversification rates through time where positive (negative) values indicate an increase (decrease) and log‐transformed clade species richness. Shaded areas represent 95% confidence intervals of fitted generalised linear models with quasibinomial error distribution and logit link function weighted for clade richness.

Posterior simulations further supported the ‘historical’ models, with observed proportional assemblage richness falling within the 95% CI for 21 (‘historical global rate’) and 25 (‘historical variable rate’) of 25 clades. In contrast, the ‘non‐historical’ model reliably predicted assemblage richness in only 16 out of 25 clades (Figure 3c). Across clades, the maximum proportion of species that co‐occur increases with clade age and mean terminal branch length and declines with clade richness (Figure 4, Figures S4 and S6). There was no effect of phylogenetic imbalance nor family‐level ρ on the maximum proportion of co‐occurring species. These patterns were also predicted when we simulated assemblages under both the ‘historical global rate’ and ‘historical variable rate’ models. In contrast, the ‘non‐historical’ null model predicts little variation in proportional assemblage richness (Figure 3c) and no correlation with phylogenetic metrics (Figure 4). Using assemblages of mean rather than maximum species reduces the variation in richness observed across clades but does not qualitatively alter our conclusions (Figure S6).

## 4 Discussion

The history of speciation, extinction and colonisation is increasingly recognised as a fundamental driver of contemporary biodiversity (Mittelbach and Schemske 2015; Ricklefs 1987). By expanding upon a dynamic model of community assembly that explicitly incorporates these processes, we demonstrate that past speciation events have left a persistent legacy on the richness of passerine bird assemblages.

Central to our model is the assumption that lineages arise in allopatry and that the subsequent accumulation of species in sympatry depends on colonisation, a process widely supported across both animal and plant taxa (Coyne and Orr 2004; Coyne and Price 2000; Olivares et al. 2025; Phillimore et al. 2008). Focussing on the most species‐rich assemblage within each bird family, we estimate a mean lag‐time (i.e., 1/γ) of approximately 8 Myr between the onset of speciation and a lineage's incorporation into a focal assemblage. Notably, this assembly rate is comparable to the macroevolutionary pace of speciation itself: the average per‐lineage waiting time between speciation events leading to extant birds is approximately 6.25 Myr (i.e., 1/0.16 species Myr^−1^) (Jetz et al. 2012). While the stochastic nature of colonisation will result in some species attaining sympatry rapidly, this parity in the rate at which species are produced and subsequently incorporated into assemblages suggests that colonisation is a major bottleneck in the build‐up of local richness.

Critically, we show that these slow rates of colonisation mean that differences in speciation history across clades lead to predictable variation in the maximum levels of sympatric diversity attained by different avian families. A higher proportion of species co‐occur in older clades as these contain older species that have had more time to accumulate in sympatry. Conversely, while larger clades have more speciose assemblages in absolute terms, they exhibit lower proportional sympatry because they are often characterised by more recent, rapid diversification and are thus dominated by younger lineages that have not yet overcome the barriers to co‐occurrence.

One potential explanation for this persistent legacy of speciation is that adaptation to spatially non‐overlapping habitats (environmental filtering) can prevent co‐occurrence (Marx et al. 2017; Pinto‐Ledezma et al. 2019). However, this is unlikely to explain the patterns observed here. Broad‐scale environmental filtering should theoretically exclude young and old species with equal probability, effectively decoupling species age from the probability of sympatry. Under such a scenario, our dynamic model would have inferred rapid turnover, rather than the low rates of colonisation and local extinction that we recovered (Pigot and Etienne 2015). The ‘speciation legacy’ we observe therefore points toward a process where barriers to sympatry are systematically overcome, but only over macroevolutionary timescales.

While birds can expand ranges rapidly across continuous landscapes, the geographic features that initiate speciation—such as the Andes or habitat breaks between Amazonian and Atlantic forests—can continue to enforce spatial isolation for millions of years (Mikkelsen et al. 2025; Naka and Brumfield 2018; Pérez‐Emán 2005; Weir and Price 2011). Even when physical barriers are breached, competition for ecological resources and reproductive interference can further constrain the transition to secondary sympatry (Anderson and Weir 2021; Pigot et al. 2018; Weir and Price 2011). In birds, complete reproductive isolation typically requires millions of years to evolve (Price 2008; Price and Bouvier 2002). Premature secondary contact can either cause the formation of hybrid zones that maintain abutting (i.e., parapatric) ranges or fusion back into a single lineage (Cooney et al. 2017; Tobias et al. 2020). Furthermore, competitive exclusion can maintain parapatry unless lineages have diverged in resource‐related traits. In the ovenbirds and woodcreepers analysed here, for example, sympatry is attained more rapidly among sister species with divergent beak morphologies (Pigot and Tobias 2013). However, because most lineages have highly conserved ecomorphology, the expected waiting time to secondary sympatry frequently exceeds the age of the species themselves, resulting in essentially indefinite spatial exclusion (Pigot and Tobias 2013). Ultimately, these interactive constraints, involving physical, reproductive and ecological barriers, mean that the timing of past speciation events remains a fundamental limit on contemporary species richness.

This interpretation of speciation legacies depends on whether local richness is ecologically bounded. If assemblage richness is unbounded—increasing as species expand into novel niche space (Harmon and Harrison 2015)—the slow rates for colonisation we infer represent a real constraint in the build‐up of sympatric richness. Conversely, if richness is regulated around a dynamic equilibrium (Rabosky and Hurlbert 2015), the importance of species age might instead reflect a ‘priority effect.’ In this bounded scenario, older species are more likely to be present not because colonisation is inherently slow, but because these species arrived first and pre‐empted finite ecological opportunities, effectively barring the subsequent establishment of younger lineages (Reijenga et al. 2021; Stroud et al. 2024). Resolving these competing interpretations is a critical next step that will require moving beyond constant‐rate models to consider diversity‐dependent dynamics whereby rates of colonisation and local extinction vary according to local species richness or niche similarity (Valente et al. 2015). Nevertheless, we contend that a simple incumbency model is unlikely to account for our results. Priority effects are expected to operate most strongly among closely related lineages that occupy similar niches within the same assemblage (Fukami 2015). Thus, while priority effects can explain why older species within a clade are more likely to occur in sympatry, incumbency does not, by itself, explain why entire families composed of younger species should exhibit proportionally lower sympatry. Consequently, a model of slow colonisation driven by dispersal limitation and the time‐dependent weakening of competitive constraints is more consistent with the speciation legacies observed here than a model of strict ecological limits.

While ultimately requiring further analysis with models that explicitly incorporate biotic interactions, our results suggest that patterns previously put forward as evidence for ecological limits could also be explained by speciation history. For example, Weir (2006) found that Amazonian bird clades with stronger diversification slowdowns (measured by the γ‐statistic) contained more speciose assemblages, interpreting this as evidence that local niche saturation inhibits range expansions required for speciation (Weir 2006). However, the γ‐statistic is sensitive to clade size and, as we have shown here, the proportion of co‐occurring species declines with clade richness even in the absence of diversity‐dependence due to the lag‐time between speciation and assemblage colonisation (Figure S8). Indeed, using a metric independent of clade size (ρ) (Pigot et al. 2010), we find no relationship between diversification slowdowns and the proportion of species that co‐occur locally (Figure 4). While our results do not address the possibility that ecological limits have caused a slowdown in diversification (Rabosky and Hurlbert 2015), they show that patterns in assemblage species richness should be evaluated against models incorporating the geographic context of speciation before being interpreted as evidence of ecological limits.

A second line of evidence for ecological limits involves Lineage‐through‐time (LTT) plots of ‘community phylogenies’, which track how the number of phylogenetic branches leading to the species currently present in an assemblage has accumulated over time (Price et al. 2014). Just as LTT plots have been used to detect slowdowns in diversification (Etienne et al. 2012; Etienne and Haegeman 2012), it has been suggested that slowdowns in community LTT plots could indicate a declining colonisation rate due to the filling of local niche space (Price et al. 2014; Weir 2006). However, we hypothesised that such slowdowns could also be a dual artefact of phylogenetic sampling and the historical legacy of speciation. First, because a community phylogeny is essentially a global tree pruned to include only the species currently present in an assemblage, it is subject to the same mathematical bias as incomplete taxonomic sampling, which is known to create artificial slowdowns in diversification rates (Cusimano and Renner 2010). Second, this effect is compounded by the speciation legacy itself: if the transition to sympatry is slow, recently diverged lineages are unlikely to co‐occur locally, further hollowing out the recent branches of the community phylogeny.

**FIGURE 5:**
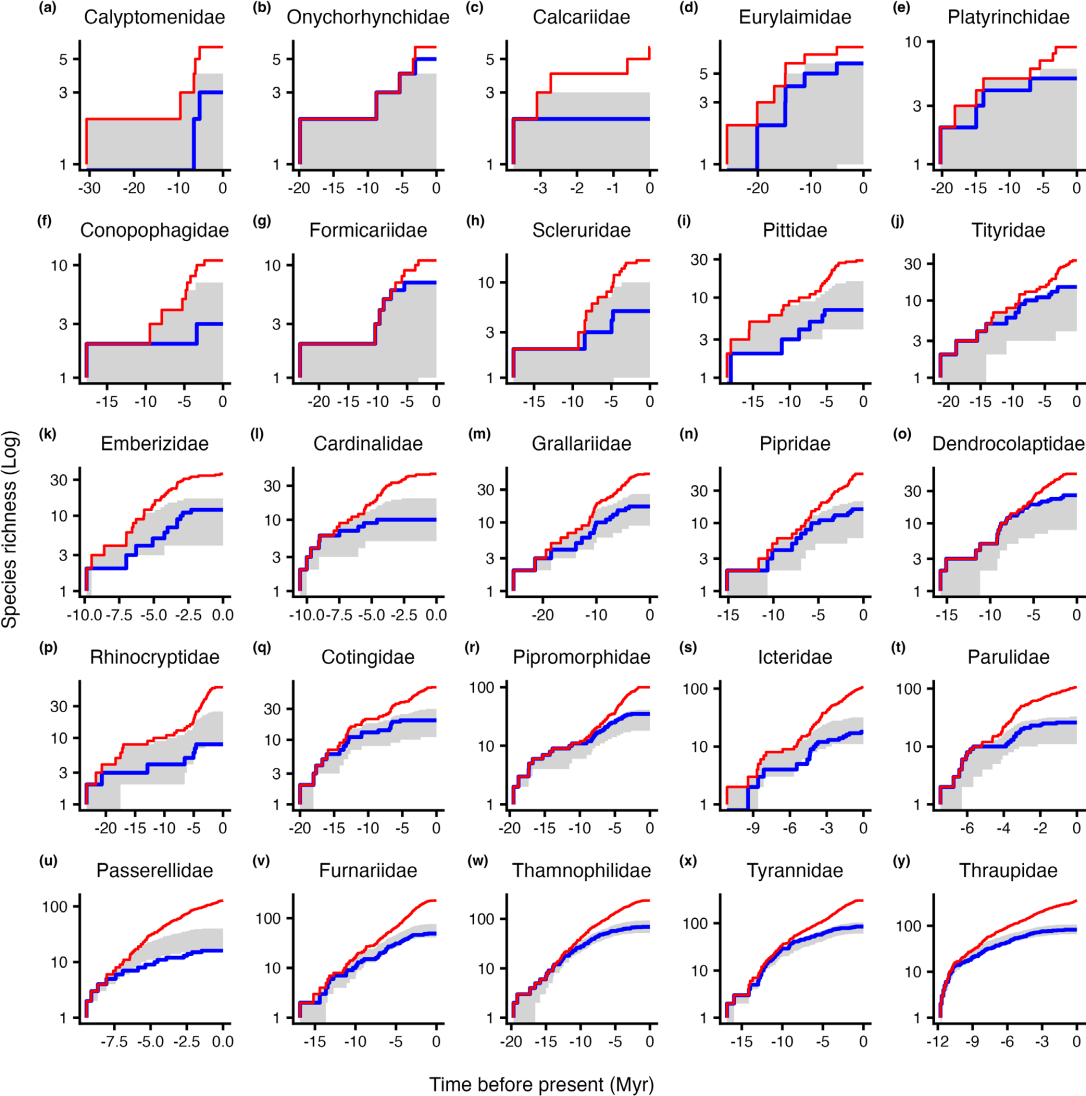
Clade and assemblage lineage‐through‐time (LTT) plots showing the accumulation of lineages through time within each family (red) and assemblage (blue, i.e., grid cell of maximum richness) compared to model predictions. Shaded areas correspond to the 95% confidence intervals of a rank envelope (Mrkvička et al. 2017) for the assemblage LTT expected under the ‘historical global rate’ model (*n* = 2500 replicate simulations). Families are ordered from smallest (Calyptomenidae, 6 species) to largest (Thraupidae, 354 species).

To test this, we performed a post hoc analysis comparing observed community LTT plots for each passerine family against our three constant‐rate models using both a rank envelope test (Mrkvička et al. 2017; Murrell 2018) and the 95% confidence interval in expected ρ (see Supporting Informations). While avian community phylogenies consistently showed temporal slowdowns (Figure 5), these patterns were indistinguishable from our ‘non‐historical’ null model in 92% of clades (except *Calyptomenidae* and *Tyrannidae*) (Figure S7). Because the non‐historical null generates assemblages that are essentially random with respect to phylogeny, this suggests most observed slowdowns arise simply because of pruning the tree to sympatric species. Furthermore, when compared to our historical models, no clades consistently exhibited stronger slowdowns than expected and several families (e.g., *Emberizidae*, *Parulidae* and *Thraupidae*) showed significantly weaker slowdowns than predicted (Figure 5). These results demonstrate that the combination of phylogenetic pruning and a macroevolutionary lag in colonisation is sufficient to explain community LTT slowdowns without invoking ecological limits. Just as ‘protracted speciation’ can mimic diversification slowdowns (Etienne and Rosindell 2012), our results show that the time required for spatially isolated lineages to achieve sympatry creates a similar illusion of niche saturation.

While our analysis demonstrates that speciation history drives predictable variation in assemblage richness, several caveats remain. First, our use of coarse geographic range maps to define assemblages aligns with broad‐scale macroecological analyses but likely captures a stronger historical signal than would be found using smaller‐scale local inventories (Harmáčková and Remeš 2024). At finer spatial grains, higher rates of local extinction and temporal turnover (Dornelas et al. 2014; MacArthur and Wilson 1967) may erode the imprint of speciation history, as seen in the rapid turnover inferred for small Alpine plant assemblages (Marx et al. 2017). Second, even at the spatial scale we use, much of the variation in maximum sympatric diversity observed across bird families remains unexplained, likely reflecting clade‐specific differences in dispersal ability, environmental filtering or biotic interactions. For example, while Woodcreepers (*Dendrocolaptidae*, *n* = 26 of 54 species) and Royal flycatchers (*Onychorhynchidae*, *n* = 5 of 6 species) exhibit significantly higher sympatric richness than expected based on their speciation history, New World Sparrows (*Passerellidae*, *n* = 16 of 127 species) and Tapaculos (*Rhinocryptidae*, *n* = 8 of 58 species) fall significantly below predicted levels (Figure 3b). By identifying clades where levels of sympatry depart from that expected due to speciation history alone, our models could help identify the additional factors that accelerate or inhibit the build‐up of sympatric diversity. Ultimately, while speciation history has left an indelible signature on current biodiversity patterns, fully decoding this legacy will require more complex models that integrate interspecific interactions (Reijenga et al. 2023) and the feedbacks between local assembly and regional diversification (Rabosky 2013; Reijenga et al. 2021; Storch and Okie 2019).

## Author Contributions

Bouwe R. Reijenga, David J. Murrell and Alex L. Pigot conceived the study. Bouwe R. Reijenga and Rampal S. Etienne developed the methodology. Bouwe R. Reijenga conducted analyses and wrote the initial manuscript. All authors contributed substantially to revisions.

## Funding

Alex L. Pigot and Bouwe R. Reijenga thank the Royal Society for funding through a Royal Society Research Fellowship awarded to Alex L. Pigot and a studentship to Bouwe R. Reijenga.

## Conflicts of Interest

The authors declare no conflicts of interest.

## Supporting information


**Figure S1:** Predicted versus maximum empirical sympatric diversity across the families at the 2600 km^2^ scale. The figure illustrates the strong positive link between assemblage and clade diversity. The predicted assemblage diversity results from a random‐draw model in which the empirical proportions of maximum co‐occurring diversity of each of the 25 families are randomly redistributed across the 25 families. This process is repeated a thousand times and assemblage diversity is then calculated by multiplying the randomised proportions with the total species richness of the family. 95% Confidence intervals are calculated from the 1000 replicates. The random‐draw null model captures 25 out of 25 families.
**Figure S2:** Maximum assemblage diversity compared between grid cell sizes. The species richness of the grid cells with the maximum richness for all clades is compared to the richness for our two grid cell sizes (96 vs. 2600 km^2^). The line through the origin indicates where species richness across scales is equal. The plot shows that richness across scales is marginally different for most families.
**Figure S3:** Observed and predicted proportional richness of passerine clades at the 96 km^2^ scale. This figure is the equivalent of Figure 2, but at the 96 km^2^ instead of the 2600 km^2^ scale. For respectively (a–c) the predicted proportional assemblage diversity according to the historical variable rate, the historical global rate and the non‐historical models is plotted against the empirical proportions. Bars represent the 95% confidence intervals of the proportional richness of the community recovered under 2500 simulations. Colours indicate if empirical proportions fall within (blue) or outside (red) of the confidence intervals. For the non‐historical model 25 out of 25 empirical proportion fall within the 95% confidence interval, whereas 23 out of 25 fall within the confidence intervals of the historical variable rate model and 19 out of 25 for the historical global rate model.
**Figure S4:** Relationship between evolutionary history and proportional assemblage diversity at the 96 km^2^ scale. This figure is the equivalent of Figure 3, but at the 96 km^2^ instead of the 2600 km^2^ scale. Colours and rows denote the empirical relationships for all clades (orange) and relationships simulated under the historical variable rate (yellow), historical global rate (blue) and the non‐historical (dark blue) models. For the simulation scenarios the mean proportion across 2500 simulations are plotted. Columns represent the five metrics representing evolutionary history: The crown age of the clade, phylogenetic imbalance (Colless' index), mean branch length of the extant species, ρ a measure of temporal change in diversification rates through time where positive (negative) values indicate an increase (decrease) and log‐transformed clade species richness. Shaded areas represent 95% confidence intervals.
**Table S1:** Parameter estimates for the historical global and variable rate models. Parameter estimates are given for the entire dataset and family‐level phylogenies when conditioning on communities of at least 1 species (cond = 1) and when no conditioning is applied (cond = 0). 95% confidence intervals were calculated via parametric bootstrapping across 100 simulations.
**Figure S5:** Observed relative mean and predicted proportional richness and diversification rate of passerine clades. The figure is equivalent to Figure 2, but for each clade an assemblage of mean richness is used instead of maximum richness. For respectively (a–c) the predicted proportional assemblage diversity according to the historical variable rate, the historical global rate and non‐historical models are plotted against the empirical proportions. The line through the origin shows where the empirical equals the simulated proportion. Bars represent the 95% confidence intervals of the proportional richness of the assemblages recovered under 2500 simulations. Colours indicate if empirical proportions fall within (blue) or outside (red) of the confidence intervals. For respectively the historical variable rate (AIC = 1204.018), the historical global (AIC = 1162.246) rate and the non‐historical model (AIC = 1205.494) (a) 25 out of 25, (b) 23 out of 25 and (c) 21 out of 25 fall within the confidence intervals for sympatric diversity.
**Figure S6:** Relationship between evolutionary history and proportional mean assemblage diversity. The figure is equivalent to Figure 3, but for each clade an assemblage of mean richness is used instead of maximum richness. Colours and rows denote the empirical relationships for all clades (orange) and relationships simulated under the historical variable rate (yellow), historical global rate (blue) and the non‐historical (dark blue) models. For the simulation scenarios the mean proportion across 2500 simulations are plotted. Columns represent the five metrics representing evolutionary history: the crown age of the clade, phylogenetic imbalance (Colless' index), mean branch length of the extant species, ρ which is a measure of temporal change in diversification rates through time where positive (negative) values indicate an increase (decrease) and log‐transformed clade species richness. Fitted Generalised Linear Models are shown and shaded areas represent 95% confidence intervals.
**Figure S7:** Comparisons of simulated and empirical diversification rate patterns. Results represent the same set of simulations as shown in Figure 2 at the 2600 km^2^ scale. On the *x*‐axis of each plot the empirical ρ is shown, calculated by pruning the family‐level phylogenies of any species that do not occur in the assemblage of highest sympatric species richness. Panels show the estimated ρ across 2500 phylogenies pruned by removing the species absent from the simulated assemblages for respectively (a) the historical variable rate model (22/25), (b) the historical global rate model (20/25) and (c) the non‐historical model (23/25). Negative values of ρ indicate slowdowns in diversification rate through time, whereas positive values indicate increases.
**Figure S8:** The relationship between the γ‐statistic and assemblage richness. Relationships are shown between the assemblage‐level (2600 km^2^) γ‐statistic, where negative values indicate slowdowns in diversification rate. To calculate γ, phylogenies were pruned to only include species that were present in the geographical cell with the highest species richness (*x*‐axis) of the respective families for the empirical data, or the simulated presence for the simulation scenarios. The scenarios from left to right, top to bottom, represent (a) the empirical relationship, (b) historical variable rate model, (c) the historical global rate model and (d) the non‐historical model. Trends shown are significant linear regression fits.

## Data Availability

Data and code are available on figshare at https://doi.org/10.25446/oxford.27025531.
